# Post-Translational Modification of PTEN Protein: Quantity and Activity

**DOI:** 10.3389/or.2024.1430237

**Published:** 2024-07-31

**Authors:** Xiao Li, Pu Yang, Xiaoli Hou, Shaoping Ji

**Affiliations:** ^1^ Department of Basic Medicine, Zhengzhou Shuqing Medical College, Zhengzhou, Henan, China; ^2^ Department of Biochemistry and Molecular Biology, Medical School, Henan University, Kaifeng, Henan, China

**Keywords:** PTEN, phosphorylation, acetylation, ubiquitination, SUMOylation

## Abstract

Post-translational modifications play crucial roles in regulating protein functions and stabilities. PTEN is a critical tumor suppressor involved in regulating cellular proliferation, survival, and migration processes. However, dysregulation of PTEN is common in various human cancers. PTEN stability and activation/suppression have been extensively studied in the context of tumorigenesis through inhibition of the PI3K/AKT signaling pathway. PTEN undergoes various post-translational modifications, primarily including phosphorylation, acetylation, ubiquitination, SUMOylation, neddylation, and oxidation, which finely tune its activity and stability. Generally, phosphorylation modulates PTEN activity through its lipid phosphatase function, leading to altered power of the signaling pathways. Acetylation influences PTEN protein stability and degradation rate. SUMOylation has been implicated in PTEN localization and interactions with other proteins, affecting its overall function. Neddylation, as a novel modification of PTEN, is a key regulatory mechanism in the loss of tumor suppressor function of PTEN. Although current therapeutic approaches focus primarily on inhibiting PI3 kinase, understanding the post-translational modifications of PTEN could help provide new therapeutic strategies that can restore PTEN’s role in PIP3-dependent tumors. The present review summarizes the major recent developments in the regulation of PTEN protein level and activity. We expect that these insights will contribute to better understanding of this critical tumor suppressor and its potential implications for cancer therapy in the future.

## Introduction

Post-translational modifications (PTMs) such as phosphorylation, acetylation, ubiquitination, SUMOylation (small ubiquitin-like modifier), neddylation, and oxidation are key regulatory mechanisms in the fine control of protein functions, stabilities, and cell localizations [1]. The phosphatase and tensin-homolog in chromosome 10 (PTEN) is a crucial tumor suppressor protein that negatively regulates the phosphoinositide 3-kinase (PI3K) signaling pathway. Upon activation of PI3K, PTEN converts phosphatidylinositol (3,4,5)-triphosphate (PIP3) to phosphatidylinositol (4,5)-biphosphate (PIP2) and attenuates the downstream AKT activity. Dysregulation of PTEN could lead to overactivation of PI3K/AKT signaling and promotion of tumorigenesis [2]. The activity and stability of PTEN are regulated by various PTMs. Deletion of PTEN activity is also involved in tumorigenesis and progression in many types of cancers [3].

Phosphorylation is one of the most extensively studied PTMs that modulates PTEN activity. Several phosphorylation sites have been identified on PTEN, including serine, threonine, and tyrosine residues. Phosphorylation can either activate or inhibit PTEN functions depending on the specific site and context. 

Acetylation is another PTM that influences PTEN protein stability, activity, or subcellular location. Acetylation of specific lysine residues on PTEN have been shown to enhance its stability and tumor-suppressive functions. Protein acetylation is regulated by lysine acetyltransferase and deacetylase. PTEN is associated with acetyltransferase in a growth-factor-dependent manner; this association leads to acetylation of the PTEN catalytic domains K125 and K128, resulting in functional inactivation of PTEN. Understanding the regulatory mechanisms of PTEN acetylation is therefore critical for developing effective cancer therapies [4, 5].

The ubiquitination of PTEN is influenced by multiple factors, including its phosphorylation status and interactions with specific regulatory proteins. Ubiquitination and proteasomal degradation are critical processes involved in PTEN stability and/or translocation. PTEN can be targeted by ubiquitin ligases, which attach ubiquitin molecules to PTEN, marking it for proteasomal degradation [6]. Dysregulation of PTEN ubiquitination disrupts its tumor-suppressive function. When PTEN is monoubiquitinated on the conservative residues K13 and K289, it can localize in the nucleus more easily and retain its tumor suppressor activity. However, when it is polyubiquitinated, it is targeted for degradation in the cytosol, leading to loss of its tumor suppressor activity [7]. Interestingly, the conformational state of PTEN also impacts its susceptibility to ubiquitination, as the open and unphosphorylated conformation is more easily ubiquitinated and degraded [8].

SUMOylation is another covalent modification that attaches small ubiquitin-like modifier proteins to the PTEN proteins. SUMOylation influences PTEN stability, cellular localization, and protein–protein interactions [9]. The process of SUMOylation necessitates the involvement of an E1-activating enzyme, an E2-conjugating enzyme, and an E3 SUMO ligase, although it can occur even in the absence of the latter [10]. SUMOylation of PTEN has been shown to promote its nuclear localization and enhance its interactions with specific transcription factors, leading to altered gene expression patterns [11]. The process by which neural-precursor-cell-expressed developmentally downregulated 8 (NEDD8) is specifically covalently attached to the substrate protein is called neddylation, and PTEN is a new substrate for neddylation modification [12]. PTEN contains cysteine residues in the active sites, which can be regulated in response to oxidative stress.

Loss of PTEN function leads to upregulation of PI3K signaling, which is widely regarded as one of the most common factors in cancer development. In addition, PTEN is involved in the tolerances of cancer cells to chemotherapy drugs and tumor metastasis, together with various other conditions [13]. Recent studies have suggested that PTEN protein levels, activities, and stabilities are tightly regulated by a broad array of PTMs; disruption of these modifications can perturb PTEN functions and contribute to the development and progression of cancers [14]. Further investigation is therefore required to elucidate the precise molecular mechanisms underlying these modifications to develop potential therapeutic strategies for PTEN-related cancers.

## Diversity Modifications in the PTEN Protein

**FIGURE 1:**
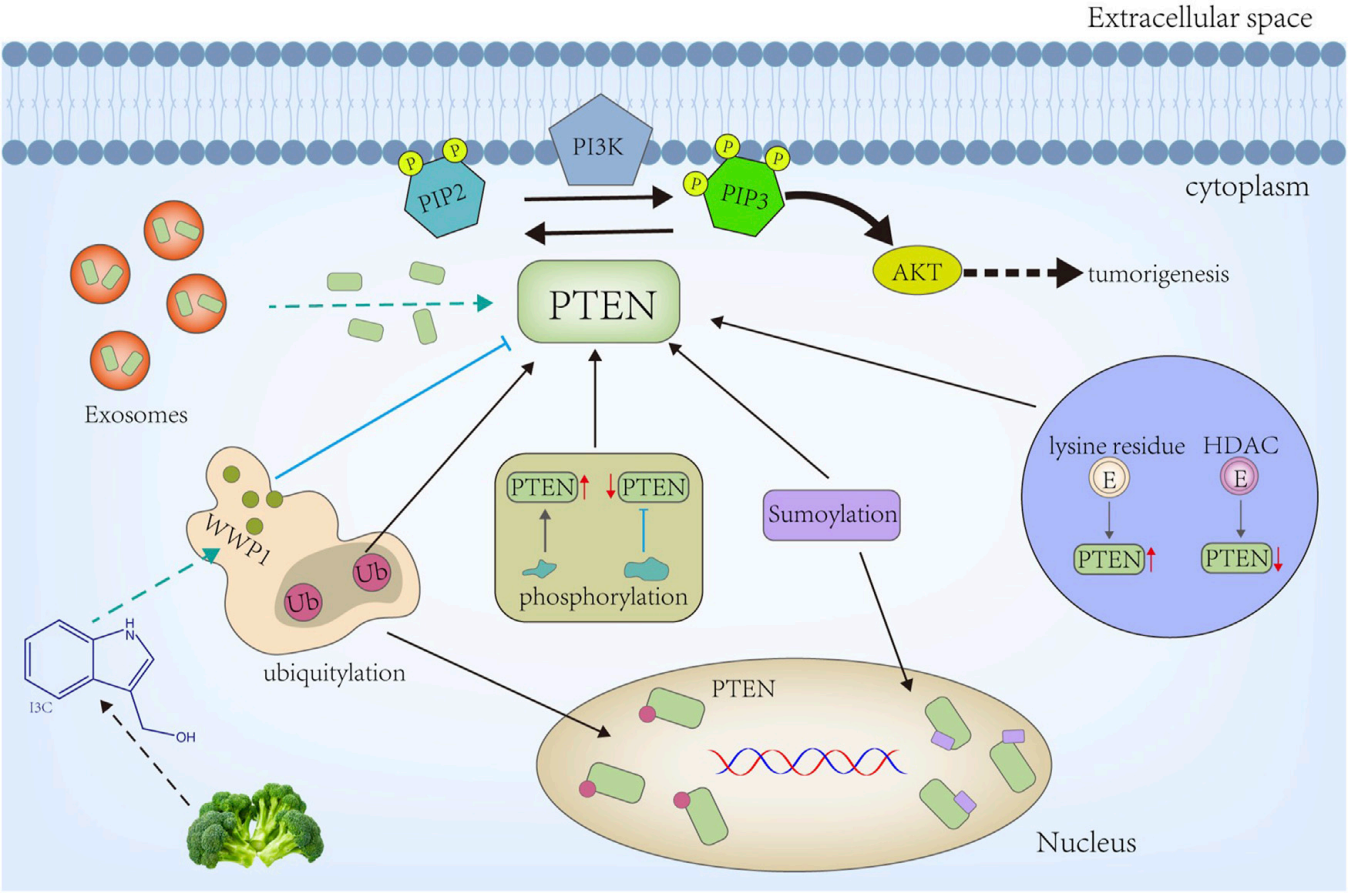
PTEN is recruited into the membrane from the cytoplasm, where it primarily dephosphorylates PIP3 (IP3) to PIP2 and represses the IP3→AKT signaling activity to facilitate tumorigenesis. Diverse modifications occur on the PTEN protein layer, from altering the protein amount to its subcellular location and activity. Among these, phosphorylation and acetylation can have different effects on PTEN activity depending on the modified residues. Ubiquitylation and SUMOylation can alter PTEN’s subcellular location or degradation. Conversely, deubiquitylation stabilizes the PTEN protein. Furthermore, recent studies have revealed that exosomes can transport PTEN proteins directly to other cells, including PTEN-deficient cells.

PTEN undergoes various PTMs, including phosphorylation, acetylation, ubiquitination, SUMOylation, neddylation, and oxidation. These modifications dynamically regulate PTEN’s subcellular localization, stability, enzymatic activity, and protein–protein interactions [1]. Protein modifications in PTEN can fine-tune its signaling pathways and impact diverse cellular processes (Figure 1).

## Phosphorylation

Phosphorylation plays a key role in the regulation of PTEN, and such phosphorylation at multiple residues regulates the activity, localization, stability, and protein interactions [15]. One of the most extensively studied phosphorylation sites in PTEN is serine 380 (Ser380). Glycogen synthetase kinase 3β mediated phosphorylation of PTEN at Ser380 promotes PTEN association with the plasma membrane, resulting in increased dephosphorylation of PIP3 and activation of PI3K/AKT signaling [16]. Conversely, Sema4D promotes PTEN dephosphorylation at Ser380 in the hippocampus and enhances PTEN phosphatase activity [17]. In addition to Ser380, other phosphorylation sites in PTEN, such as T366, T240, and Ser229, have been identified and implicated in modulating its activities and interactions with other proteins [18]. The fibroblast growth factor receptor 2 mediated phosphorylation of PTEN at T240 (pY240-PTEN) protects cells from DNA damage by promoting DNA repair. The nuclear localization and chromatin binding of pY240-PTEN has been shown to increase after treatment with ionizing radiation and promote DNA repair, indicating that pY240-PTEN downregulates PTEN antitumor activity [19].

PTEN protein phosphatase activity can dephosphorylate phosphoglycerate kinase 1 and inhibit its phosphorylation, thereby inhibiting glycolysis [20]. Cellular exposure to recombinant transcription factors or protease activated receptor 2 agonist peptide (PAR2-AP) can activate PTEN. PAR2 activation releases PTEN from the membrane-associated guanylate kinase with inverted configuration (MAGI complex) [21]. At the protein level, melanin II increases PTEN through the melanocortin 1 receptor, mediating reduction of PTEN phosphorylation and inducing PTEN expression upregulation. As a result, upregulation of PTEN inhibits the AKT/nuclear factor κ B signaling pathway, repressing migration, invasion, and colony-forming capability in the melanoma cells [22]. Phosphorylation regulation of PTEN is a complex process that impacts its tumor suppressor functions and cellular processes associated with growth, survival, and proliferation. The balance between phosphorylation and dephosphorylation events at specific residues determines PTEN’s activity and its crucial role in maintaining cellular homeostasis.

## Acetylation

Acetylation is a common PTM that regulates PTEN; it occurs at specific lysine residues and influences the activity, stability, and cellular localization of PTEN [23]. One of the extensively studied acetylation sites in PTEN is K125. PTEN interacts with the acetyltransferase p300/CBP-related factor (PCAF) in a manner dependent on growth factors. The acetylation of K125 by PCAF has been demonstrated to augment both PTEN stability and phosphatase activity [5].

Additionally, some histone deacetylase (HDAC) inhibitors have been shown to enhance the translocation of PTEN membrane or promote the activation and expression of PTEN. The translocation of the PTEN membrane is enhanced by trichostatin A or octylaniline hydroxamic acid. PTEN’s C-tail interacts with another part of the protein, blocking its membrane translocation and activation. Acetylation of PTEN at K163 induces conformational changes, facilitating activation and membrane translocation [24]. In addition, valproic acid inhibitor can enhance PTEN expression and block the AKT/mTOR pathway [25]. A negative correlation also exists between the transfer-related protein 1 (MTA1) and PTEN, where MTA1 knockdown could enhance the acetylation of PTEN. Resveratrol, a dietary compound found in grapes and wine, promotes acetylation and reactivation of PTEN by inhibiting the MTA1/HDAC complex [26].

## Ubiquitination

Ubiquitination plays a crucial role in the regulation of PTEN; when PTEN is subjected to ubiquitination by several E3 ubiquitin ligases, such as NEDD4-1, WW-domain-containing E3 ubiquitin protein ligase 2 (WWP2), BRCA1-associated protein 1 (BAP1), ovarian tumor protease deubiquitination enzyme 5 (OTUD5), and ovarian-tumor-domain-containing protein 3 (OTUD3). Ubiquitination of PTEN can have dual effects on its stability and activity. Inhibiting PTEN ubiquitination mediated by E3 ligases through drugs or gene targeting can effectively unleash PTEN’s anticancer activity and inhibit tumorigenesis [27].

NEDD4-1 was the first identified PTEN E3 ubiquitin ligase; it recognizes specific motifs in PTEN and catalyzes the addition of ubiquitin chains to PTEN, marking it for proteasomal degradation [28]. WWP2-mediated ubiquitination of PTEN produces different outcomes [29]; instead of promoting degradation, WWP2-mediated ubiquitination has been reported to stabilize PTEN [30]. Studies have shown that deubiquitination is related to the PTEN protein level. The BAP1 deubiquitinating enzyme plays a crucial role in deubiquitination of PTEN; overexpression of BAP1 can upregulate PTEN protein levels. BAP1 can physically combine with and deubiquitinate PTEN, thereby inhibiting the ubiquitin-mediated degradation of PTEN and stabilizing the PTEN protein [31].

USP10 significantly increases the phosphatase activity of PTEN via deubiquitination and stabilization of PTEN [32]. Differentially, Gao et al. found that once USP25 was knocked down, the cell proliferation increased, apoptosis decreased, and PI3K/AKT signaling pathway activated by the PTEN protein level decreased [33].

OTUD5 can deubiquitinate and stabilize PTEN. However, miR-652-3p can target and repress OTUD5 expression, thereby inhibiting its deubiquitination effect on PTEN and downregulating PTEN protein levels [34]. Moreover, OTUD3 depletion results in cell transformation, cancer metastasis induction, and AKT signaling pathway activation. Interestingly, OTUD3 transgenic mice showed elevated levels of PTEN and were less prone to developing tumors. Thus, the OTUD3-PTEN axis could be a promising target for cancer prevention and treatment [35, 36].

The ubiquitination and protein levels of PTEN are influenced by the isoleucine levels in the cytoplasm. Isoleucine promotes PTEN entry into the nucleus, increases PTEN protein level, and inhibits PTEN ubiquitination to maintain PTEN stability in the nucleus, where PTEN stabilizes the genome. Therefore, isoleucine may achieve antitumor activity by influencing the level of PTEN accumulation in the nucleus instead of transcriptional activation [37]. 

The balance between ubiquitination and deubiquitination thus determines the stability and activity of PTEN.

## Focus on WWP1 and PTEN Activities

As an E3 ubiquitin ligase, WWP1 recognizes and binds to PTEN, facilitating transfer of ubiquitinated PTEN to the proteasome for degradation [38]. 

The ubiquitination and degradation of PTEN by WWP1 reduces the PTEN protein levels, impairing its tumor suppressor function. WWP1 variants have been shown to cause cancer in many patients without PTEN deficiency. In a recent study, Jiang et al. identified two functional gain variants of WWP1, namely K740N and N745S, which have the ability to activate WWP1 E3 ligase while promoting ubiquitination and degradation of PTEN to activate the PI3K pathway [39]. WWP1 blocked PTEN activity by polyubiquitination, blocking of PTEN dimerization, and membrane translocation. Indole-3-methanol (I3C) derived from cruciferous vegetables was discovered to inhibit WWP1 activity effectively [40]. Similarly, Wang et al. found that the expressions of WWP1 mRNA and proteins increased in thyroid papillary carcinoma cells; knocking down WWP1 could upregulate PTEN expression and inhibit cancer [41]. PTEN membrane localization can be restored by genetic or chemical inhibition of WWP1, and tumor growth can be inhibited *in vitro* and *in vivo* in cooperation with PI3K inhibitors [42].

## SUMOylation

SUMOylation governs an array of processes including transcriptional regulation, subcellular localization, protein stability, protein–protein interactions, and protein–DNA interactions [43]. 

Recent research has revealed that PTEN is modified by SUMO1 at the K254 and K266 residues, especially the latter. This modification plays a key role in preventing activation of the PI3K/AKT signaling pathway and anchoring PTEN to the plasma membrane [44]. The conjugation of SUMO to K254 has been suggested to enhance its nuclear localization. This finding highlights the importance of the interactions between PTEN and SUMO, revealing how PTEN localization is controlled at the molecular level [45]. Recently, new discoveries have been reported on the SUMOylation of PTEN, revealing that PTEN’s K266 and K289 are required for its antiviral activity against the vesicular stomatitis virus [46]. In addition, K254 SUMOylation does not affect PTEN’s ability against PI3K but is involved in the DNA damage response [45].

SUMOylation of PTEN modulates its protein–protein interactions. SUMOylation has been shown to enhance the interactions between PTEN and other proteins, such as p53, resulting in synergistic tumor suppressor effects [47]. By activating PTEN SUMO, the protein inhibitor of the activated STAT xα (PIASxα) reduces PTEN ubiquitination to promote PTEN protein stability and upregulate the protein level. However, the mutation of PTEN’s SUMO1 binding site can neutralize the effects of PIASxα on the half-life of the PTEN protein. These findings provide important insights into the regulation of PTEN protein stability and role of PIASxα in this process [48].

Dysregulation of PTEN SUMOylation has been observed in various cancers, leading to altered PTEN stability, subcellular distribution, and functional interactions. Investigating the regulation of PTEN SUMOylation provides insights into the intricate mechanisms controlling PTEN’s tumor suppressor activities and involvement in various cellular processes, particularly in cancer.

## Neddylation

NEDD8 is the molecule that is most similar to ubiquitin and can bind to PTEN. Xie et al. showed that under high glucose conditions, neddylation promotes PTEN entry into the nucleus and enhances the PI3K/AKT signaling pathway to promote tumorigenesis; neddylation also controls PTEN subcellular localization when glucose is excessive or lacking [49].

## Oxidation

Oxidation of PTEN results in its dissociation from the plasma membrane; this suggests that PTEN oxidation not only affects its activity but also regulates its cellular localization, effectively removing it from the main site of lipid phosphatase activity [50]. Peroxidoredoxin can prevent the oxidation of PTEN under benign oxidative stress through direct interactions, thereby maintaining and promoting the tumor suppressor function of PTEN [51]. In streptozotocin (STZ)-induced diabetes, STZ toxicity is mediated by cytokine and oxidative stress, leading to β cell damage. Under mechanical conditions, these factors inhibit AKT activation by upregulating PTEN expression and p-PTEN levels, thereby inducing β cell apoptosis [52].

## Regulation of PTEN via Exosomes

Exosomes are small extracellular vesicles released by various cell types [53]; they have emerged as important regulators of intercellular communications since they can transfer functional biomolecules, including proteins, lipids, and nucleic acids, between cells to influence cellular processes [54]. Recent studies have shown that exosomes can also modulate PTEN activity through different mechanisms. 

It has been identified that exosomes regulate PTEN functions through the transfer of miRNAs. For example, miR-152-3p in the mesenchymal stem cell (MSC)-derived exosomes has been shown to upregulate PTEN expressions in the recipient cells, promoting wound healing in diabetic foot ulcers [55]. Other studies have shown that miR-144-5p directly targets PTEN and promotes PTEN expression, leading to apoptosis of vascular endothelial cells in intracranial aneurysms [56]. Interestingly, exosomes from human umbilical cord MSCs were found to contain lncRNA PTENP1, which stabilizes PTEN expression by competitively binding to miR-10a-5p in the U87 cells [57].

In contrast, some miRNAs in the exosomes have been shown to downregulate PTEN expressions in recipient cells, leading to activation of the PI3K/AKT pathway. Some examples of these are miR-18b-5p [58], miR-148-3p [59], miR-21 [60], miR-181b-5p [61], miR-205 [62], and miR-934 [63]. 

Moreover, exosomes can directly modulate PTEN activity through transfer of the PTEN protein itself. The NEDD4 family interacting protein 1 (Ndfip1) is a molecular regulator of PTEN secretion and impacts non-cellular autonomous PTEN activity. PTEN secretion in the exocrine system requires Ndfip1. K13 in PTEN is required for transport of the PTEN exosomes [64]. Similarly, exosomes secreted by cells with PTEN mutations or loss may contribute to PTEN inactivation in the recipient cells. These exosomes may contain altered miRNA profiles or lack functional PTEN proteins, promoting PTEN deficiencies in the recipient cells and oncogenic signaling thereof.

## Protein–Protein Interactions

The activities and subcellular localizations of the PTEN proteins are regulated through interactions with specific proteins [65]. In addition to its interactions with AKT, PTEN can form complexes with other proteins like the discoid homologous region (PDZ-domain) proteins, which help stabilize PTEN and regulate its cellular localization. Scaffold proteins like MAGI-1b, MAGI-2, and MAGI-3 interact with PTEN’s PDZ binding domain and recruit PTEN to the membrane complex, where PTEN dephosphorylates PIP3 [66–68]. A recent study revealed that the β-suppressor scaffold protein is the upstream signal regulator of PTEN; here, PTEN membrane localization and lipid phosphatase activity increase through direct contact [69].

The maintenance of chromosome stability is ensured by the nuclear PTEN through its interactions with the centromere protein-C [70]. The interactions between PTEN and specific partners also affect PTEN’s subcellular localizations. For instance, ubiquitin-conjugating enzymes interact with PTEN and modulate its translocation to the nucleus [71]. Moreover, ataxia telangiectasia mutated gene phosphorylates PTEN and transfers it to the cytoplasm, where it interacts with p53 to regulate cell-cycle progression [72].

## Perspectives

PTEN is the second most-mutated tumor suppressor after p53 in cancer and is associated with many human diseases. Understanding and studying the PTMs of PTEN can help in the discovery of new therapeutic strategies to restore PTEN activities and protein levels in PIP3-dependent tumors (Table 1). PIK3CA is the catalytic subunit of the PI3KsIA type; mutations in the PIK3CA gene promote cell carcinogenesis through the PI3K/AKT pathway [73]. G protein signal transduction 12 binds to PTEN through the PDZ domain, upregulating the phosphorylation and SUMOylation of PTEN as well as inhibiting oral cancer development [74]. PTEN deficiency promotes gemcitabine efficacy in cancer by modulating the phosphorylation of protein phosphatase 2A [75]. PTEN inactivation mediated by overexpression of E3 ubiquitin ligase causes glioblastoma cells to adapt to the PTEN deficiency, thereby developing resistance to epidermal growth factor receptor tyrosine kinase inhibitors and immunotherapies [76].

**TABLE 1:** Post-translational modifications of PTEN and possible intervention strategies.

PTM	Residue	Biological outcome	Possible intervention
Phosphorylation	Ser380 [17] T366 and T240 [18]	Reduced PTEN	Casein kinase 2 inhibition
Acetylation	K163 [24]	Translocation to plasma membrane and increased phosphatase activity	HDAC inhibition
Ubiquitination	K342 and K344	Reduced PTEN activity	
WWP1	—	Blocking PTEN dimerization and membrane translocation	Indole 3 carbinol
SUMOylation	K266 and K254 [45]	Membrane and increased phosphatase activity	SUMO-specific protease inhibition or activation of specific E3 ligase
Exosomes	—	Upregulation of PTEN expression	—

## Conclusion

As an important tumor suppressor, PTEN effectively inhibits the activity of the PI3K/AKT carcinogenic signaling pathway through its lipid phosphatase activity. In addition, PTEN achieves inhibitory effects on tumor cells through other mechanisms without depending on the PI3K/AKT pathway. It is well known that PTEN dysregulation is extensively involved in tumor occurrence and development. Therefore, the regulatory mechanisms of PTEN capacity have become the focus in the field of tumor biology research. A comprehensive understanding of the molecular and cellular mechanisms underlying the regulation of PTEN activity and protein level is crucial for clinical practitioners and researchers. In addition, this provides new research directions and therapeutic avenues for the application of PTEN in anticancer therapies.
